# Incidental Cardiac Metastasis in Breast Carcinoma

**DOI:** 10.3390/diagnostics16010071

**Published:** 2025-12-25

**Authors:** Yaomin Chen, Haibo Wang, Zhiyan Fu, Ellen Elizabeth Connor

**Affiliations:** Department of Pathology, Louisiana State University Health Sciences Center, New Orleans, LA 70112, USA; ychen3@lsuhsc.edu (Y.C.); hwang6@lsuhsc.edu (H.W.)

**Keywords:** breast carcinoma, cardiac metastasis, autopsy, histological examination

## Abstract

**Background and Clinical Significance**: Metastatic breast cancer is a major global health burden, with common metastatic sites including the bones, lungs, liver, and brain. Cardiac metastasis is rare and often clinically silent, leading to underdiagnosis. Recognizing cardiac involvement, even when asymptomatic, is important for understanding the full extent of disease and ensuring optimal patient care. **Case Presentation**: We report the case of a woman with advanced breast carcinoma who showed no clinical or imaging evidence of cardiac involvement throughout the course of her illness. Following her death from progressive metastatic disease, an autopsy revealed metastatic carcinoma infiltrating the myocardium and epicardium without gross cardiac abnormalities. Histological and immunohistochemical analysis confirmed the tumor’s origin from breast carcinoma. **Conclusions**: This case illustrates the potential for clinically occult cardiac metastasis in breast cancer and underscores the importance of pathological examination in detecting hidden metastatic sites. The absence of cardiac symptoms or imaging abnormalities highlights the diagnostic challenge of this rare manifestation and the need for greater awareness in managing advanced malignancies.

## 1. Introduction

Breast cancer is the most frequently diagnosed malignancy and remains the leading cause of cancer-related mortality among women worldwide [1]. When metastatic, it commonly spreads to the bones, lungs, liver, and brain [2]. While cardiac metastasis is considered rare and often overlooked in routine clinical evaluations, postmortem studies have demonstrated that cardiac involvement is more frequent than previously appreciated [3]. These metastases may affect any layer of the heart, including the pericardium, myocardium, or endocardium, and can result in complications such as arrhythmias, heart failure, or pericardial effusion [4,5]. Despite the potential severity, most cardiac metastases remain clinically silent, without overt symptoms, and are often identified incidentally during imaging or autopsy examinations [6]. The under-recognition of cardiac metastasis in patients with advanced breast cancer can pose a diagnostic challenge. However, its identification holds significant clinical importance. Awareness of this potential involvement is essential, particularly in patients presenting with unexplained cardiac symptoms or during the evaluation of disseminated malignancies. This report presents a case of cardiac metastasis secondary to breast carcinoma, identified at autopsy in a patient who exhibited no clinical signs suggestive of cardiac involvement. It highlights the silent nature of cardiac metastases and the role of pathology in uncovering such occult metastases and emphasizes the importance of maintaining awareness of cardiac involvement in disseminated malignancies for comprehensive evaluation and management.

## 2. Case Presentation

**Figure 1:**
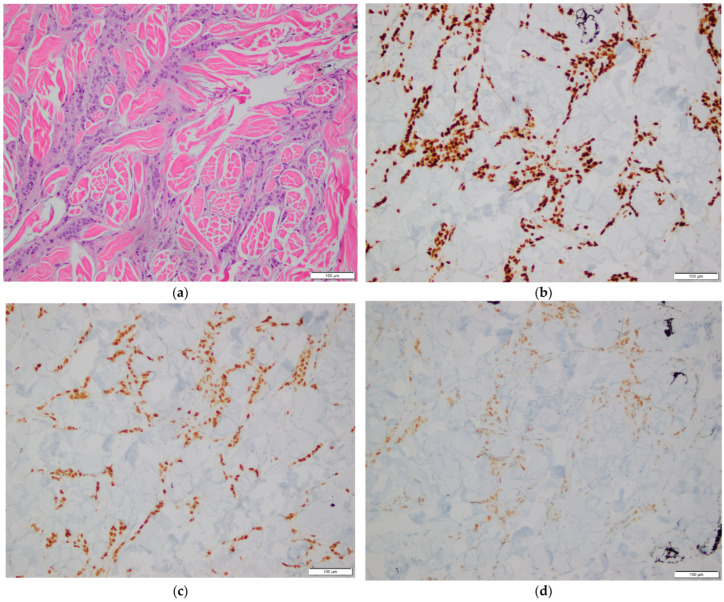
Histopathological examination of breast tissue. (**a**) Hematoxylin and eosin (H&E) stain showing invasive breast carcinoma. 200× magnification; (**b**–**d**) Immunohistochemical stains showing tumor cell positivity for GATA3 (**b**), ER (**c**), and PR (**d**). 200× magnification.

A 50-year-old African American woman with a six-month history of a right breast mass was diagnosed with invasive breast carcinoma of no special type (NST) (Figure 1) in July 2020. She received systemic chemotherapy and adjuvant radiation therapy, followed by a right mastectomy in February 2021, after which she experienced a period without clinical evidence of disease. In October 2022, she presented with shortness of breath, and imaging revealed a large right-sided pleural effusion and diffuse sclerotic lesions involving the vertebral bodies, ribs, and sternum, concerning for metastatic disease. Recurrent breast cancer was confirmed pathologically in November 2022. In 2023 and 2024, she had multiple admissions for worsening dyspnea and emesis, requiring serial thoracenteses and placement of a pleural catheter for recurrent right-sided pleural effusions. In December 2024, she was admitted with persistent nausea, vomiting, and progressive shortness of breath. Chest radiograph and computed tomography (CT) revealed bilateral pleural effusions without any cardiac findings. Further cardiac evaluation, including transthoracic echocardiogram (TTE) and electrocardiogram (ECG), showed no abnormalities. During hospitalization, she developed pneumonia caused by methicillin-susceptible *Staphylococcus aureus* (MSSA) and systemic *Candida glabrata* fungemia. Despite broad-spectrum antibiotics, antifungal therapy, and intensive supportive measures, her condition deteriorated, and she died shortly thereafter.

**Figure 2:**
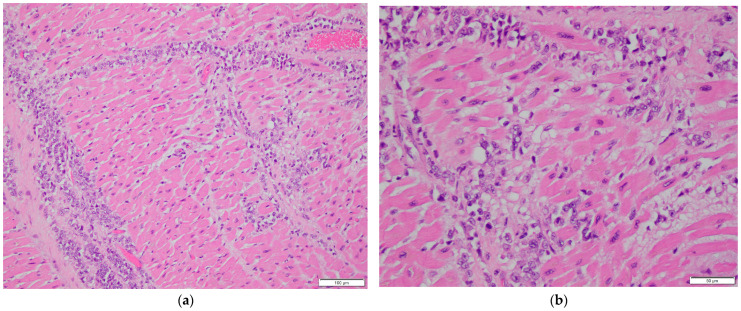

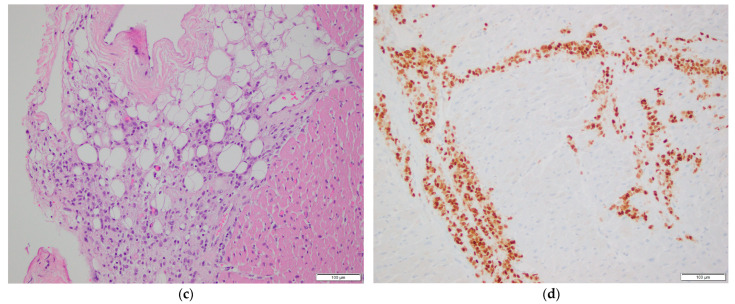

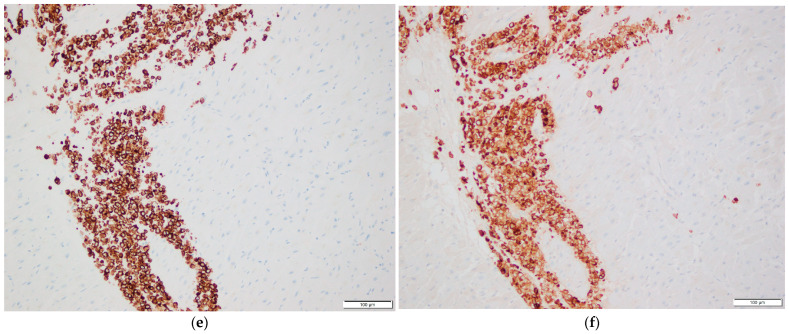

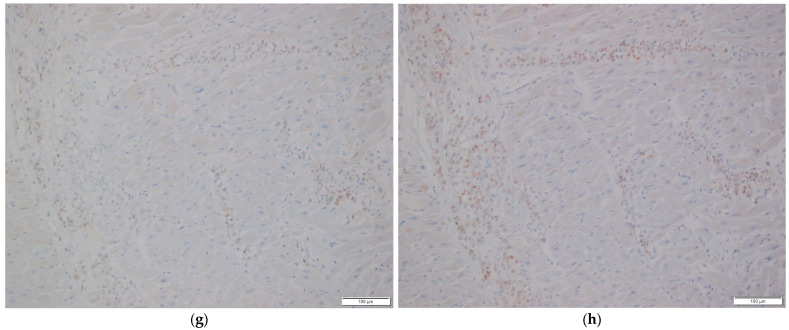
Histopathological examination of cardiac tissue. (**a**,**b**) Myocardial involvement by metastatic breast carcinoma. H&E stain, 200× magnification in (**a**), 400× magnification in (**b**). (**c**) Epicardial involvement by metastatic breast carcinoma. H&E stain, 200× magnification. (**d**–**h**) Immunohistochemical stains of myocardial tissue showing tumor cell positivity for GATA3 (**d**), CAM5.2 (**e**), EMA (**f**), ER (**g**), and PR (**h**). 200× magnification.

A complete autopsy was performed. Gross examination of the heart revealed no abnormalities. The pericardium was intact with no evidence of effusion. The heart weighed 200 g (normal range: 210–350 g). The right and left ventricular walls measured 0.3 cm and 1.0 cm, respectively (normal ranges: 0.25–0.5 cm and 1.0–1.5 cm). The interventricular septum measured 1.0 cm (normal range: 0.9–1.2 cm) (All normal reference ranges are based on gender and body weight). Cardiac chambers were of normal size and configuration. There were no grossly visible tumors or nodular lesions on the epicardial surface, within the cardiac chambers, or in the myocardium. However, histologic examination revealed multifocal infiltration of the myocardium and epicardial adipose tissue in both ventricles and the interventricular septum by malignant epithelial cells. These cells exhibited pleomorphic, cleared-out nuclei with clumped chromatin, prominent nucleoli, and frequent mitotic figures (Figure 2a–c), morphologically similar to the cancer cells identified in the previously excised breast tissue. Immunohistochemical stains showed that the tumor cells were positive for GATA3, CAM5.2, epithelial membrane antigen (EMA), estrogen receptor (ER), and progesterone receptor (PR), consistent with metastatic breast carcinoma (Figure 2d–h). The immunoprofile matched the phenotype of the patient’s primary breast tumor. Additional metastatic foci were identified in both lungs, liver, kidneys, appendix, uterus, and right periadrenal adipose tissue, indicating disseminated metastatic disease at the time of death.

## 3. Discussion

Breast cancer remains one of the most commonly diagnosed malignancies globally. It is a leading cause of cancer-related mortality among women, with incidence rates continuing to rise in many parts of the world due to population aging and increasing exposure to risk factors. Advances in early detection and treatment have improved survival rates for localized disease; however, metastatic breast cancer remains largely incurable and is associated with a poor prognosis. Among the sites of distant metastases, cardiac involvement is under-recognized, despite autopsy series reporting incidence rates ranging from 10% to 12% in patients with advanced disease [3]. This discrepancy between clinical detection and autopsy findings may reflect the silent nature of cardiac metastases and/or the limitations of routine diagnostic approaches.

When breast carcinoma spreads to the heart, the pericardium is most frequently involved, followed by the myocardium and, less commonly, the endocardium [7]. Myocardial involvement, as demonstrated in the present case, is particularly insidious due to its deep anatomical location, minimal outward distortion of cardiac morphology, and typically silent clinical behavior. These characteristics often allow such metastases to go undetected through routine imaging or clinical evaluation. Consequently, patients may present without specific cardiac symptoms, and the disease may only be identified postmortem. The absence of clinical signs does not indicate the absence of pathological impact; rather, it underscores the importance of targeted investigations—particularly in patients with advanced malignancies—and highlights the critical role of histopathological evaluation in uncovering otherwise occult metastases and determining the true extent of disease dissemination.

In this report, we present a case of widely disseminated metastatic invasive breast carcinoma in a 50-year-old woman, with incidental myocardial and epicardial involvement discovered at autopsy. The patient’s final clinical course was dominated by gastrointestinal complaints and respiratory distress, which were subsequently complicated by MSSA pneumonia and *Candida glabrata* fungemia. There were no overt clinical signs of cardiac metastasis, and imaging studies, TTE and ECG, did not raise suspicion of cardiac involvement. Gross examination of the heart at autopsy appeared unremarkable, without evidence of masses, effusions, or structural compromise. This observation further emphasizes the diagnostic difficulty in identifying cardiac metastases without dedicated investigations or high clinical suspicion.

Histologic sections of the heart, however, revealed multifocal infiltration of the myocardium and epicardial adipose tissue by malignant epithelial cells. These cells displayed characteristic cytologic features of high-grade carcinoma, including enlarged, cleared-out nuclei, coarse clumped chromatin, prominent nucleoli, and frequent mitotic figures, resembling those seen in the patient’s primary breast carcinoma. Immunohistochemical staining confirmed the tumor’s origin, with neoplastic cells demonstrating strong nuclear positivity for GATA3, a marker frequently expressed in breast carcinomas. ER and PR expression were positive but notably weak, which may reflect reduced antigenicity of these hormone receptors due to the prolonged cold ischemia time commonly encountered in autopsy specimens.

Evidence from the literature suggests that cardiac metastases from breast carcinoma are typically accompanied by the involvement of other organs, either preceding or occurring concurrently with cardiac dissemination [8,9,10,11,12]. This reflects the systemic nature of metastatic breast cancer and its tendency to spread widely in advanced stages. In our patient, this pattern was clearly demonstrated, as metastatic deposits were also identified in the lungs, liver, kidneys, appendix, uterus, and right periadrenal adipose tissue. The presence of such widespread involvement not only confirms the advanced stage of the disease but also reflects the aggressive biological behavior of the tumor. These findings indicate the importance of considering the heart as a potential site of metastasis in patients with extensive multi-organ involvement, even in the absence of clinical cardiac symptoms.

The presence of incidental cardiac metastasis raises important considerations regarding its underlying pathophysiological mechanisms and potential clinical implications. Several pathways have been proposed to explain the metastatic spread of cancer cells to the heart, which include hematogenous dissemination, lymphatic invasion, transvenous extension via the superior vena cava, and direct invasion from adjacent anatomical structures [3,13,14]. In breast cancer, lymphatic and hematogenous routes are considered the predominant pathways [7]. The heart’s rich blood supply and extensive lymphatic network make it a theoretically susceptible target for metastatic seeding, although the actual incidence of clinically apparent metastases remains low. The absence of cardiac symptoms in our case, despite myocardial and epicardial infiltration, reflects the heart’s capacity to accommodate metastatic foci without immediate functional compromise, at least until the disease burden becomes hemodynamically or electrically significant.

Clinically, although cardiac metastases are frequently asymptomatic, they may present with a broad spectrum of manifestations depending on the location and extent of involvement. These can include dyspnea, chest pain, palpitations, arrhythmias, conduction abnormalities, heart failure, or pericardial effusion leading to tamponade [4,5]. In rare instances, sudden cardiac death may occur. These manifestations are often misattributed to other common complications in cancer patients, including anemia, pulmonary embolism, pleural effusion, or treatment-related cardiotoxicity, leading to underdiagnosis. In our patient, the presenting symptoms included shortness of breath. Although a cardiac etiology cannot be entirely excluded, normal TTE and ECG findings suggest that the symptoms were more likely attributable to pleural effusion and possible underlying pulmonary pathology.

When cardiac symptoms arise in patients with known malignancy, echocardiography remains the first-line diagnostic modality for evaluating cardiac metastases. It offers rapid bedside evaluation and can detect pericardial effusion, chamber compression, and intra- or pericardial masses, although its utility is limited by suboptimal acoustic windows and operator dependency [15,16]. CT provides superior spatial resolution and can quickly define lesion extent and its relationship to adjacent cardiac structures, but it exposes patients to radiation and contrast agents [17]. Cardiac magnetic resonance imaging (MRI) is currently considered the most accurate technique for evaluating cardiac metastases, as it reliably characterizes neoplastic infiltration based on tissue properties [18]. However, the use of cardiac MRI in routine oncologic evaluation is limited by cost, availability, and patient factors such as claustrophobia or the presence of non-MRI-compatible implants. In the absence of high clinical suspicion, cardiac metastases are rarely investigated with these diagnostic modalities. As a result, the true incidence of cardiac involvement in cancer may be under-estimated. As illustrated by our case, even extensive myocardial and epicardial infiltration can remain undetected during life and only be discovered during postmortem examination, which highlights the value of autopsy in elucidating the full burden of metastatic disease.

While the incidental discovery of cardiac involvement may not alter the clinical course or prognosis in patients with advanced disease, early recognition in selected patients may guide clinical management. For instance, detecting cardiac metastases may prompt more intensive cardiac monitoring, particularly for arrhythmias or heart failure symptoms. It may also affect the selection or modification of systemic therapies to minimize further cardiac compromise. In palliative settings, awareness of cardiac metastases can influence symptom management strategies, such as the use of diuretics for effusion or antiarrhythmics for conduction disturbances. In rare cases where cardiac obstruction causes significant symptoms, surgical intervention may be considered if the anticipated benefits outweigh the operative risks [3,19]. In addition to surgical approaches, other therapeutic options such as cardiac-directed radiotherapy and chemotherapy may offer palliative benefits. Radiotherapy has been used to relieve symptoms related to mass effect, while certain chemotherapeutic agents with activity against the primary malignancy may reduce cardiac tumor burden [20,21]. The feasibility and effectiveness of these interventions depend on tumor histology, patient performance status, and overall prognosis. Therefore, identifying cardiac metastases, even when not treatable with curative intent, enables a more targeted and individualized approach to patient care.

This case contributes to the growing body of evidence suggesting that cardiac metastases, though rare, should not be overlooked as a diagnostic consideration in the context of advanced breast cancer. Moreover, it highlights the critical role of postmortem examination in detecting clinically silent metastatic disease, thereby contributing to a more comprehensive understanding of cancer dissemination patterns. As cancer therapies continue to improve survival, recognizing less typical metastatic sites becomes increasingly important—not only for guiding clinical decisions but also for improving the delivery of palliative care.

## 4. Conclusions

Metastatic involvement of the heart by breast carcinoma is rare but clinically significant, often remaining undetected during life due to its asymptomatic nature and the limitations of current diagnostic modalities. In such cases, histologic evaluation, typically through postmortem examination, plays a crucial role in establishing the diagnosis. Identifying clinically silent cardiac involvement has important implications for accurate staging, understanding metastatic patterns, and guiding clinical management in patients with disseminated malignancy. The case we presented is particularly notable for several distinguishing features. Despite diffuse infiltration of both ventricles and the interventricular septum by breast cancer cells, there were no associated symptoms, electrocardiographic changes, or imaging abnormalities, even in the terminal stage. Moreover, the heart appeared grossly unremarkable at autopsy, and the metastases were detected only microscopically, emphasizing the importance of routine histologic sampling. Detailed immunohistochemical analysis confirmed the breast origin of the cancer cells. This report underscores the diagnostic challenges of clinically silent cardiac metastases, highlights the critical role of pathology in uncovering the true extent of disease, and emphasizes the importance of increased clinical awareness of cardiac involvement in cancer. Future research should focus on enhancing diagnostic modalities and developing more tailored management strategies for cardiac metastases in cancer patients, with the ultimate goal of improving outcomes and quality of life.

## Data Availability

The original contributions presented in this study are included in the article. Further inquiries can be directed to the corresponding authors.
